# Pygmy Rice Rat as Potential Host of Castelo dos Sonhos Hantavirus

**DOI:** 10.3201/eid1708.101547

**Published:** 2011-08

**Authors:** Elizabeth S. Travassos da Rosa, Daniele B. A. Medeiros, Márcio R.T. Nunes, Darlene B. Simith, Armando de Souza Pereira, Mauro R. Elkhoury, Marília Lavocat, Aparecido A.R. Marques, Alba Valéria Via, Paulo D’Andrea, Cibele R. Bonvicino, Elba Regina S. Lemos, Pedro F.C. Vasconcelos

**Affiliations:** Author affiliations: Instituto Evandro Chagas, Ananindeua, Brazil (E.S. Travassos da Rosa, D.B.A. Medeiros, M.R.T. Nunes, D.B. Smith, A. de Souza Pereira, P.F.C. Vasconcelos);; Fundação Nacional de Saúde, Brasília, Brazil (M.R. Elkhoury);; Secretaria de Vigilância em Saúde, Brasília (M.R. Elkhoury, M. Lavocat);; Secretaria de Saúde do Estado de Mato Grosso, Cuiabá, Brazil (A.A.R. Marques, A.V. Via);; Fundação Oswaldo Cruz, Rio de Janeiro, Brazil (P. D’Andrea, C.R. Bonvicino, E.R.S. Lemos);; Instituto Nacional de Câncer, Rio de Janeiro (C.R. Bonvicino);; Universidade do Estado do Pará, Belém, Brazil (P.F.C. Vasconcelos).

**Keywords:** viruses, Castelo dos Sonhos virus, zoonoses, CASV, hantavirus, genetic characterization, Oligoryzomys utiaritensis, pygmy rice rat, Brazil, dispatch

## Abstract

To study the dynamics of wild rodent populations and identify potential hosts for hantavirus, we conducted an eco-epidemiologic study in Campo Novo do Parecis, Mato Grosso State, Brazil. We detected and genetically characterized Castelo dos Sonhos virus found in a species of pygmy rice rat (*Oligoryzomys utiaritensis*).

Hantaviruses are RNA viruses (family *Bunyaviridae*, genus *Hantavirus*) distributed worldwide. In nature, these viruses are maintained in persistently infected rodents without disease manifestation. Hantaviruses are transmitted to humans through a respiratory route, mainly by inhalation of aerosolized, virus-infected particles in rodent excreta, such as feces, saliva, or urine. Hantavirus pulmonary syndrome (HPS) was first recognized in 1993 after an outbreak of acute respiratory distress syndrome associated with Sin Nombre virus occurred in the southwestern United States (*1*). In the same year, another hantavirus (Juquitiba virus) was identified in association with HPS cases in the state of São Paulo in southeastern Brazil (*2*).

Since 1993, molecular techniques have been used to identify New World hantaviruses in samples obtained from humans suspected of having hantavirus infection throughout the Americas and from captured rodents that test seropositive for hantavirus-specific immunoglobulin (Ig) G (*3**–**5*). Most known hantaviruses associated with rodent reservoir species have been identified in this way. However, for some hantaviruses, including Castelo dos Sonhos virus (CASV), the virus–host association remains unknown.

CASV was first identified in samples from a patient with HPS in 1995 and was the first hantavirus described in the Brazilian Amazon region (*3*). We report here data obtained during an eco-epidemiologic study conducted in the municipality of Campo Novo do Parecis, Mato Grosso State in central-western Brazil (Figure 1), including the identification of a possible rodent reservoir for CASV.

**Figure 1:**
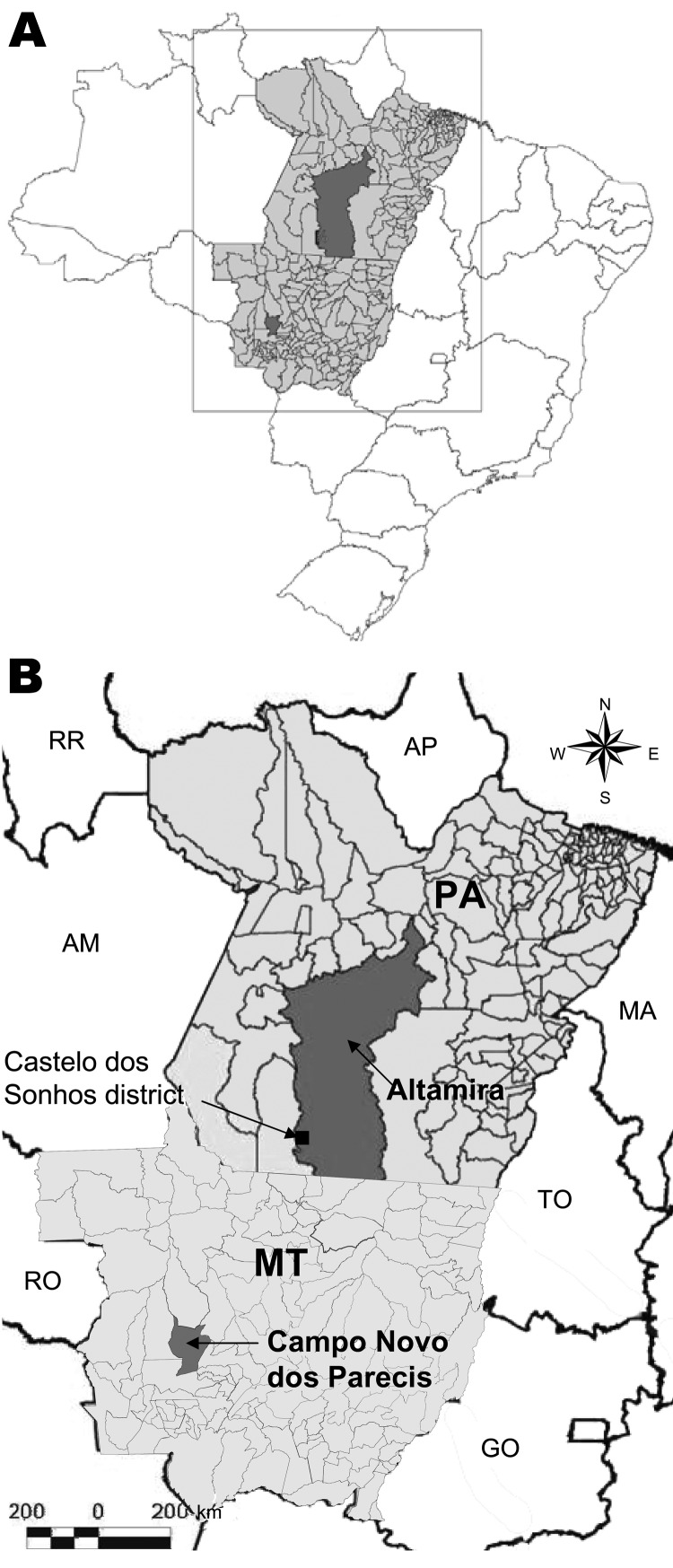
Location of Mato Grosso State, Brazil, showing the municipality of Campo Novo do Parecis where pygmy rice rats (*Oligoryzomys utiaritensis*) were found infected with Castelo dos Sonhos virus and the Castelo dos Sonhos district in the municipality of Altamira, Pará State, both locations where hantavirus pulmonary syndrome cases caused by Castelo dos Sonhos virus have been frequently found. MT, Mato Grosso State; PA, Pará State; AP, Amapá State; AM, Amazonas State; MA, Maranhão State; TO, Tocantins State; RO, Rondônia State; GO, Goiás State. Source: Laboratório de Geoprocessamento do Instituto Evandro Chagas, Secretaria de Vigilância em Saúde do Ministério da Saúde.

## The Study

During 2005 through and 2007, research was conducted in the municipality of Campo Novo do Parecis (13°40′31′′S 57°53′31′′W) to study the dynamics of wild rodent populations and prevalence of hantavirus infection. Of 459 rodents captured during the project, 89 were classified as *Olygoryzomys utiaritensis* (a species of pygmy rice rat). Blood samples obtained from the rats were serologically screened by IgG-ELISA by using the Andes virus antigen as previously described (*5*).

**Figure 2:**
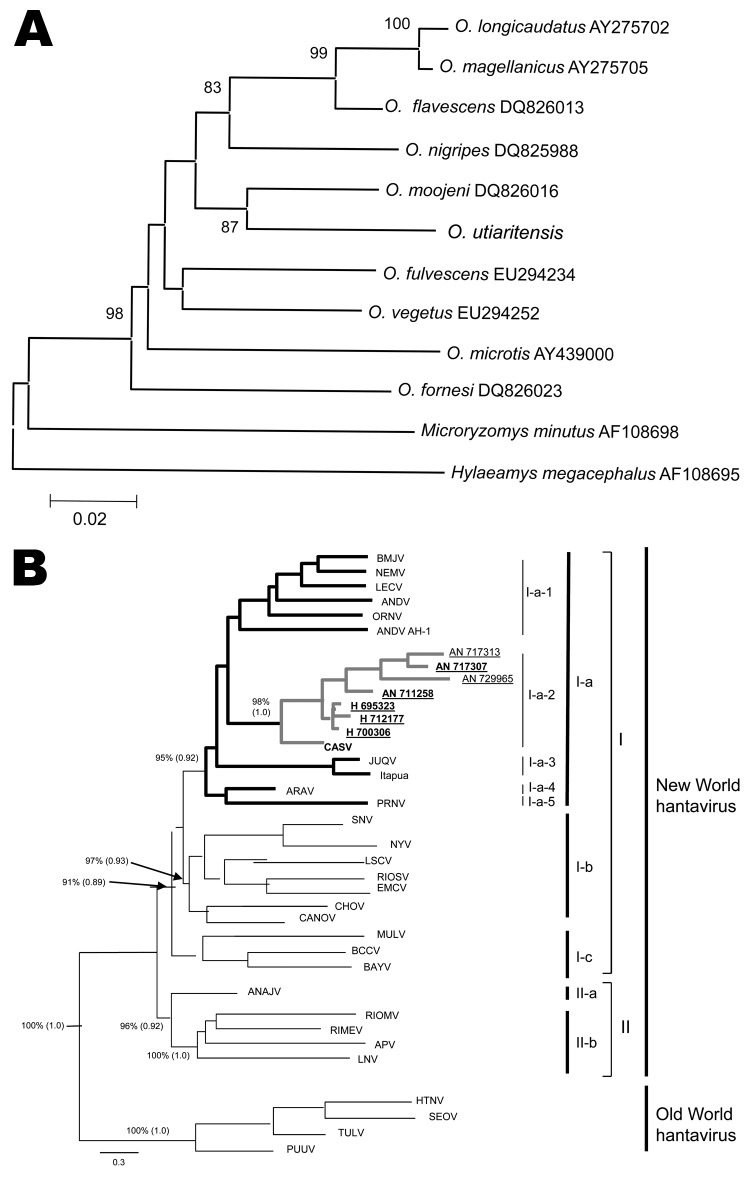
A) Phylogenetic tree constructed by using the neighbor-joining method for characterization of pygmy rice rats by using the cytochrome *b* DNA. Scale bar indicates nucleotide sequence divergence among the rodent species sequences. B) Phylogenetic tree using the maximum-likelihood (ML) and Bayesian methods based on partial nucleotide sequences of N gene obtained from pygmy rice rats captured in Campo Novo do Parecis, Mato Grosso State, Brazilian Amazon (New World major group, Group I, Clade Ia, Subclade Ia2, underlined strains and branches highlighted in **boldface**). Numbers at right indicate (left to right) subclades, clades, groups, and major groups. Green branch corresponds to Castelo dos Sonhos virus, including prototype strain, samples from pygmy rice rats, and hantavirus pulmonary syndrome–associated strains. Values over each main node placed outside and inside parentheses correspond to ML (bootstrap values) and Bayesian posterior probabilities (BPP), respectively. The arrows indicate the exact position for the bootstrap and BPP values over the main branch nodes. Scale bar indicates nucleotide sequence divergence among the hantavirus sequences. APV, Alto Paraguay virus (DQ345762); ANAJV, Anajatuba virus (DQ451829); ANDV, Andes virus (AF291702); Andes AH-1, Andes virus strain AH-1 (AF324902); ARAV: Araraquara virus (AF307325); BAYV: Bayou virus (L36929); BMJV, Bermejo virus (AF482713); BCCV, Black Creek Canal virus (L39949); CANOV, Caño Delgatito virus (DQ285566); CASV, Castelo dos Sonhos virus (AF307324); CHOV, Choclo virus (DQ285046); EMCV, El Moro Canyon virus (U54577); HTNV, Hantaan virus (NC005218); JUQV, Juquitiba virus (EF492472); LNV, Laguna Negra virus (AF005727); LECV, Lechiguanas virus (AF482714); LSCV, Limestone Canyon virus (AF307322); MULV, Muleshoe virus (U54576); NYV, New York virus (U09488); ORNV, Oran virus (AF482715); PRNV, Pergamino virus (AF482717); PUUV, Puumala virus (NC005224); RIOMV, Rio Mamore virus (U52138); RIMEV, Rio Mearim virus (DQ451828); RIOSV, Rio Segundo virus (U18100); SEOV, Seoul virus (NC005236); SNV, Sin Nombre virus (NC_005216); TULV, Tula virus (NC005227). H 695323 (HQ719468), H 712177 (HQ719467) and H 700306 (HQ719466) represent human CASV sequences obtained from patients with hantavirus pulmonary syndrome residing near the area where CASV has been recovered from pygmy rice rats.

DNA samples from the rats were isolated from liver preserved in ethanol. We amplified cytochrome *b* mitochondrial DNA (≈1,140 bp) with primers L14724 and Citb-rev by using standard PCR procedures and sequenced the samples with the same primers and an additional internal primer MVZ16 (*6*). Sequencing was performed with ABI 3130xl (Applied Biosystems, Foster City, CA, USA) automatic DNA sequencer. Kimura 2-parameter models were used for constructing neighbor-joining (NJ) dendrograms by using MEGA4 software (*7*). Confidence intervals for NJ trees were obtained by bootstrap analysis based on 2,000 replicates. Despite morphologic similarities between *O. nigripes* (black-footed pygmy rice rat) and *O. utiaritensis*, the NJ analysis showed that *O. utiaritensis* pygmy rice rats are more closely related to Moojen’s pygmy rice rat (*O. moojeni*) (7.4% Kimura 2-parameter distance estimates) than to any other *Oligoryzomys* species (Figure 2, panel A). We deposited all animal carcasses in the National Museum, Rio de Janeiro, Brazil. The record of the 4 CASV-positive animals in the National Museum and other data are shown in Table 1.

**Table 1:** Pygmy rice rat (*Oligoryzomys utiaritensis*) samples from which Castelo dos Sonhos virus genome was amplified, sequenced, and phylogenetically analyzed, Brazil*

IEC no.	SVS no.	MN no.	GenBank accession no.
AN 711258	328	MN 74939	HQ719472
AN 717307	33	MN 75063	HQ719471
AN 717313	39	MN 74965	HQ719470
AN 729965	183	MN 75064	HQ719469

*IEC, Instituto Evandro Chagas; SVS, Secretaria de Vigilância em Saúde; MN, Museu Nacional.

For hantavirus detection, reverse transcription PCR was used to synthesize cDNA with generic hantavirus primers (*3*) as previously described (*8*). N gene partial nucleotide sequences were obtained by the Sanger method (*9*) by using the same primers. At least 3 amplicons per sample were sequenced in both directions to improve coverage and confidence on results. The obtained sequences were aligned with other hantavirus sequences available in GenBank (www.ncbi.nlm.nih.gov) with ClustalW software (http://www.clustal.org) in BioEdit 5.0 (www.mbio.ncsu.edu/BioEdit/biodoc.pdf). The maximum-likelihood and Bayesian methods were implemented in PHYML and MRBAYES version 3, respectively, and used for phylogenetic reconstructions (*10**,**11*). Modeltest version 3.7 was used to determine the best nucleotide substitution model (*12*).

Four of 89 pygmy rice rat samples tested were IgG positive (AN711258, AN717313, AN717307, and AN729965) (Table 1). Partial N gene nucleotide sequences (≈400 nt; nucleotide position from 30–450 related to the CASV N gene sequence; GenBank accession no. AF307324) were obtained from lung fragments of the 4 IgG-positive rodents. Obtained sequences showed high nucleotide and amino acid homology (92.4% and 100%, respectively) with other CASV sequences in GenBank. The rodent-related strains showed 0.6% nt divergence among them, 2.1% nt sequence divergence with HPS-related strains, and 7.6% nt sequence divergence with the CASV prototype strain. These results were also confirmed by phylogenetic analysis that grouped the studied strains based on the N gene partial sequence analysis together with CASV prototype strain and local strains recovered from patients with HPS (Table 2) in the area (Figure 2, panel B).

**Table 2:** Human samples from which Castelo dos Sonhos virus genome was amplified, sequenced, and phylogenetically analyzed, Brazil*

IEC number	Patient age, y/sex	Place of infection†	GenBank accession no.
H 695323	24/M	Castelo dos Sonhos	HQ719468
H 700306	30/M	Castelo dos Sonhos	HQ719466
H 712177	36/M	Cachoeira da Serra	HQ719467

*IEC, Instituto Evandro Chagas. †All located in Pará State, Brazil.

After its isolation in 1995 from 1 patient with HPS in the Castelo dos Sonhos district of Altamira in southeast Pará State, CASV has only occasionally been detected in Pará and neighboring Mato Grosso states in Brazil’s Amazon region. However, a recent study performed among residents of 4 municipalities along interstate highway BR-163, which runs between southeastern Pará and northern Mato Grosso, has suggested continuous CASV circulation with occurrence of small outbreaks, sporadic HPS cases, and silent infections (*13*). In fact, between 1995 and 2010, a total of 72 HPS cases in southeastern Pará State were reported to the Brazilian Ministry of Heath. Although eco-epidemiologic studies were conducted as part of case investigations, none of the captured rodent species were found to be reservoirs for CASV on the basis of molecular tests used to detect the hantavirus genome.

Among Brazilian states, Mato Grosso has the fourth highest number of reported HPS cases. Cases have been associated with agricultural activities, mainly cultivation of soybean and grains, and occasionally corn and other beans. Previous studies in Mato Grosso have demonstrated the circulation of the Laguna Negra virus in the region as associated with rodents belonging to the genus *Calomys* (*14**,**15*). However, the complexity of clinical outcomes observed for patients, as well as the high case-fatality rate reported in the state of Mato Grosso and Brazilian Amazon (43.3%), were not similar to data associated with Laguna Negra infections in Paraguay (*3*), which suggests that 2 different hantaviruses related to HPS cases are co-circulating in this state.

## Conclusions

The study of dynamics of rodent populations during the period identified the rodent population that naturally occurs in the rural zone of the municipality of Campo Novo do Parecis in Mato Grosso. Lung samples from 4 pygmy rice rats showed anti-hantavirus antibodies, yielding hantavirus RNA amplification. Sequencing and phylogenetic analysis indicated those rodents were infected by CASV, suggesting this species of pygmy rice rat as a potential host of CASV. Furthermore, this rodent species was previously unknown in the Amazon region. Pygmy rice rats may be a reservoir for CASV, and this hantavirus may be responsible for HPS cases in Campo Novo do Parecis and in neighboring municipalities located in the midwestern region of Mato Grosso state in central Brazil, near the border with Pará state, and also in the Castelo dos Sonhos district of Pará state.
